# Long-Chain Acyl-CoA Synthetase 1 Role in Sepsis and Immunity: Perspectives From a Parallel Review of Public Transcriptome Datasets and of the Literature

**DOI:** 10.3389/fimmu.2019.02410

**Published:** 2019-10-18

**Authors:** Jessica Roelands, Mathieu Garand, Emily Hinchcliff, Ying Ma, Parin Shah, Mohammed Toufiq, Mohamed Alfaki, Wouter Hendrickx, Sabri Boughorbel, Darawan Rinchai, Amir Jazaeri, Davide Bedognetti, Damien Chaussabel

**Affiliations:** ^1^Sidra Medicine, Doha, Qatar; ^2^Department of Surgery, Leiden University Medical Center, Leiden, Netherlands; ^3^Department of Gynecologic Oncology and Reproductive Medicine, The University of Texas MD Anderson Cancer Center, Houston, TX, United States; ^4^Department of Melanoma Medical Oncology, The University of Texas MD Anderson Cancer Center, Houston, TX, United States

**Keywords:** sepsis, neutrophils, OMICS data, long-chain acyl-CoA synthetase, lipid metabolism

## Abstract

A potential role for the long-chain acyl-CoA synthetase family member 1 (ACSL1) in the immunobiology of sepsis was explored during a hands-on training workshop. Participants first assessed the robustness of the potential gap in biomedical knowledge identified via an initial screen of public transcriptome data and of the literature associated with ACSL1. Increase in ACSL1 transcript abundance during sepsis was confirmed in several independent datasets. Querying the ACSL1 literature also confirmed the absence of reports associating ACSL1 with sepsis. Inferences drawn from both the literature (via indirect associations) and public transcriptome data (via correlation) point to the likely participation of ACSL1 and ACSL4, another family member, in inflammasome activation in neutrophils during sepsis. Furthermore, available clinical data indicate that levels of ACSL1 and ACSL4 induction was significantly higher in fatal cases of sepsis. This denotes potential translational relevance and is consistent with involvement in pathways driving potentially deleterious systemic inflammation. Finally, while ACSL1 expression was induced in blood *in vitro* by a wide range of pathogen-derived factors as well as TNF, induction of ACSL4 appeared restricted to flagellated bacteria and pathogen-derived TLR5 agonists and IFNG. Taken together, this joint review of public literature and omics data records points to two members of the acyl-CoA synthetase family potentially playing a role in inflammasome activation in neutrophils. Translational relevance of these observations in the context of sepsis and other inflammatory conditions remain to be investigated.

## Introduction

Long-chain acyl-CoA synthetases (ACSLs) are essential enzymes that activate fatty acids (FA) by converting them to FA acyl-CoA esters. This activation is required for both synthesis of cellular lipids, such as triacylglycerol (TAG), phospholipids, and cholesterol esters as part of anabolic lipid metabolism, as well as their degradation via β-oxidation as part of catabolic lipid metabolism (1). Deregulation of FA metabolism is frequently encountered across metabolic diseases and carcinogenesis (2–4). ACSLs play a role in the pathogenesis to fatty liver disease, obesity, atherosclerosis, diabetes, neurological disorders, and specific types of cancer (4). Five ACSL family members have been described, ACSL1, ACSL3, ACSL4, ACSL5, and ACSL6, which are encoded by separate genes (5). Each of these enzymes have distinct substrate specificities, tissue specific expression patterns, and different subcellular localizations (1).

Involvement of ACSL1 in immunological processes was examined in the context of a hands-on workshop which used available public omics data as source of training material. ACSL1 was selected among a pool of gene candidates on the basis, on one hand of its upregulation in neutrophils following *in vitro* exposure to septic plasma, and on the other of the absence or limited number of literature reports linking it with neutrophil immunobiology or the pathogenesis of sepsis.

Focusing a review on a research area which has been overlooked is inherently challenging given the lack of good published reports on the topic in question. However, exploring such gaps in knowledge and attempting to define avenues for future research remains a useful endeavor. Furthermore, publicly available omics data were employed as a means to, at least in part, overcome the absence of publication on the topic being covered. Indeed, in addition to initially permitting identification of the knowledge gap this approach also provided opportunities: (1) to confirm initial observation across independent datasets, (2) to draw inferences from co-expression (guilt by association). While this review does not address the identified knowledge gap in a way a research paper would, it permits to bring such a gap to the attention of the community in a timely manner and delineates potential avenues for future investigations.

## Knowledge Gap Assessment

As described in a recent review, workshop participants for the first of three “collective omics data” (COD) training modules currently under development are tasked to assess potential gaps in biomedical knowledge for a given gene candidate (6).

**Figure 1:**
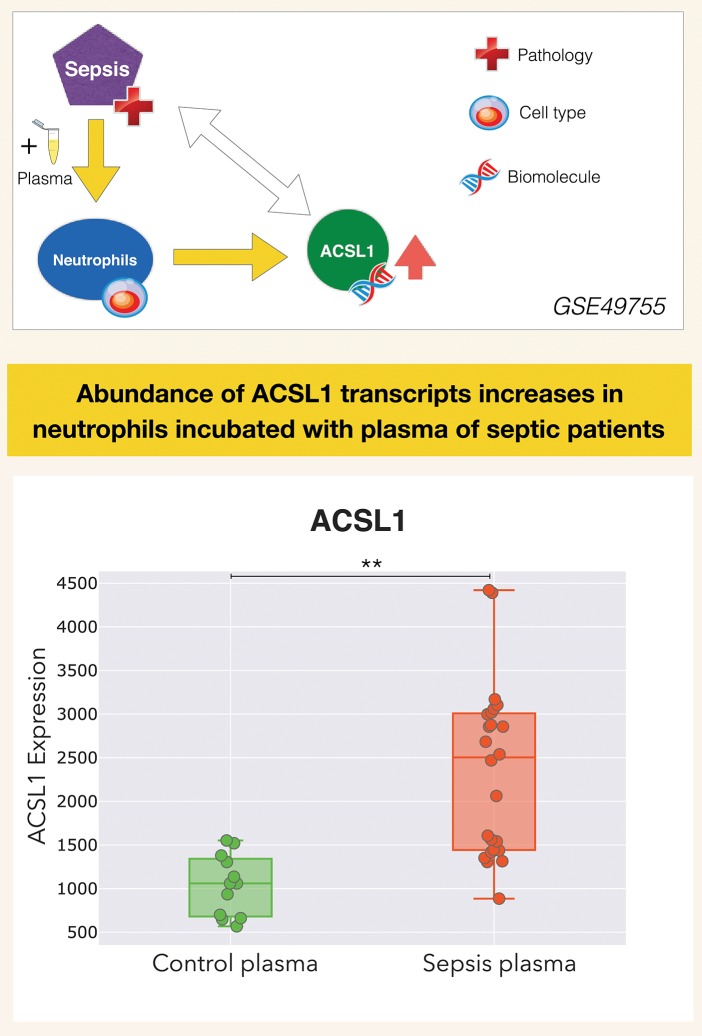
ACSL1 is upregulated by septic plasma. Abundance of ACSL1 transcripts was measured by microarray in neutrophils exposed *in vitro* for 6 h to plasma from uninfected control subjects or from patients with sepsis [from a dataset deposited in GEO by Khaenam et al. with unique identifier GSE49755 (7)]. Neutrophils were obtained from two different healthy donors, each exposed to the same set of plasma samples which consisted in plasma from 6 uninfected controls and 12 septic patients. Differences between groups were statistically significant (^**^p < 0.01), and confirmed in two independent test sets from the same study (GSE49756, GSE49757). ACSL1 profiles from all three datasets can be accessed via the GXB data browsing application: (8–10). Design elements in this and subsequent figures obtained from http://www.clker.com/ (eppendorf tube) and https://www.vecteezy.com (red cross, DNA helix, cell).

**Figure 2:**
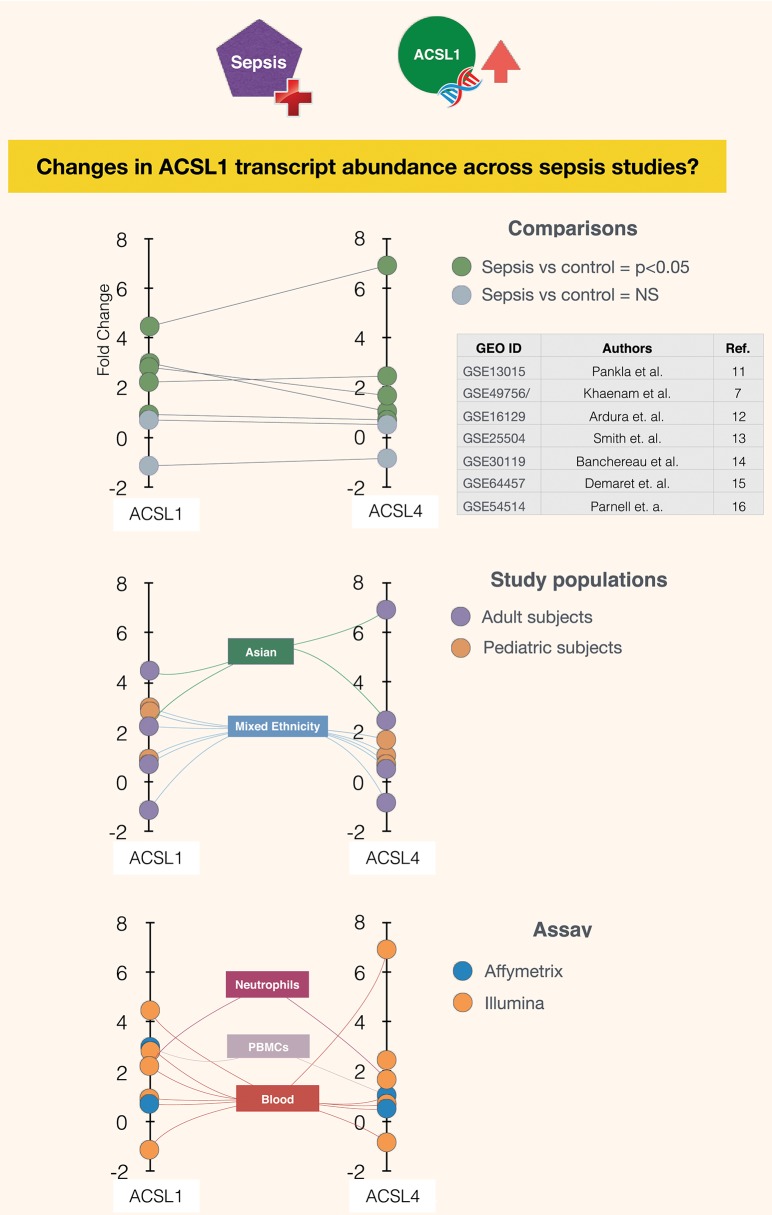
Abundance of ACSL1 and ACSL4 transcripts is significantly increased in the context of sepsis in five out of seven datasets. Fold changes in abundance of ACSL1 and ACSL4 is shown in sepsis vs. control groups across multiple datasets (7, 11–16). Each spot represents a single dataset, except for GSE49756/7 which shows averaged values for the GSE49756 and GSE49757 series and that also comprises GSE49755 (Figure 1). The position of the spot on the vertical axis indicates the corresponding fold change in abundance of ACSL1 (left) and ACSL4 (right). Spots are connected to dataset attributes (metadata). On the top panel attributes relevant to the group comparisons that were performed are shown (indicating which groups are being compared and whether increase in ACSLs abundance are statistically significant). On the middle and bottom panels additional attributes that describe the study populations and assays are shown.

It is in this context that ACSL1 was selected among a larger pool of candidate genes for which transcript abundance was elevated in neutrophil cultures exposed *in vitro* to plasma of septic patients (7) [Figure 1 and link to data browsing web application (8)]. One of the first steps consisted in evaluating the robustness of this observation by looking up ACSL1 expression profiles across datasets which were deemed suitable for this validation step (Figure 2). Suitability in this case was judged by the workshop participants based on experimental/study design (i.e., inclusion of sepsis cases and suitable controls). Importantly, in order to avoid selection bias, studies were identified for validation prior to accessing the ACSL1 transcriptional profiles to verify differential expression. Since another member of the ACSL family, ACSL4, was also among the candidates which passed our selection criteria it was decided to attempt to confirm differential expression across multiple sepsis datasets. Upregulation of ACSL1 and ACSL4 could be confirmed in both of the validation datasets deposited by Khaenam et al. (GSE49756 and GSE49757). Furthermore, significant increase in abundance for both of these members of the ACSL family were observed in four of the six independent datasets. These included whole blood and PBMCs samples collected from adult or pediatric sepsis subjects and appropriate uninfected controls, run on illumina or affymetrix array platforms and in the context of studies carried out on different continents (as indicated on Figure 2: middle and bottom panels).

With the increase in ACSL1 abundance confirmed in multiple independent sepsis datasets, the next step consisted in interrogating the literature for current knowledge about its role in this setting. Workshop participants were tasked to build a PubMed query capturing the pool of publications that constitutes the ACSL1 literature.

The query is comprised of all official names, symbols and aliases recorded for ACSL1 in the NCBI entrez gene database, OMIM and swissprot databases. The [tw] argument is added to force PubMed to perform the search as specified [(tw) = text words: thus restricting the search to text included in tiles and abstracts]:

“*ACSL1” [tw] OR “Acyl-CoA Synthetase Long Chain Family Member 1” [tw] OR “Acyl-CoA Synthetase Long Chain Family Member 1” [tw] OR “Fatty-Acid-Coenzyme A Ligase, Long-Chain 2” [tw] OR “Long-Chain Fatty-Acid-Coenzyme A Ligase 1” [tw] OR “Long-Chain-Fatty-Acid–CoA Ligase 1” [tw] OR “Long-Chain Fatty Acid-CoA Ligase 2” [tw] OR “Long-Chain Acyl-CoA Synthetase” [tw] OR “Long-Chain Acyl-CoA Synthetase 1” [tw] OR “Long-Chain Acyl-CoA Synthetase 2” [tw] OR “Lignoceroyl-CoA Synthase” [tw] OR “Palmitoyl-CoA Ligase 1” [tw] OR “Palmitoyl-CoA Ligase 2” [tw] OR “Acyl-CoA Synthetase 1” [tw] OR “LACS 1” [tw] OR “LACS 2” [tw] OR “LACS-1” [tw] OR “LACS-2” [tw] OR “FACL2” [tw] OR “FACL1” [tw] OR “LACS1” [tw] OR “LACS2” [tw] OR “ACS1” [tw] OR “LACS” [tw] OR “Fatty-Acid-Coenzyme A Ligase, Long-Chain 1” [tw] OR “Palmitoyl-CoA Ligase 1” [tw] AND (acyl [tw] OR CoA [tw] OR fatty [tw] OR synthetase [tw] OR Palmitoyl [tw] OR ligase [tw] OR ACSL1 [tw])*

This PubMed query returned a total of 625 articles as of May of 2019. The query argument, following the boolean “AND” was introduced to minimize the rate of false positives. It excluded irrelevant articles including for instance the ACSL1 alias “LACS” but referring to something entirely different, such as “Laser-Assisted Cataract Surgery” (LACS), or “Low molecular weight Aromatic Compounds” (LACs).

Next, the degree of novelty of the observation reported in Figure 1 was determined by interrogating the ACSL1 literature (i.e., assessment of a potential gap in biomedical knowledge). For this purpose, the representation in the ACSL1 literature of relevant topics was examined.

The study that served as a starting point for this review consisted in exposing neutrophils *in vitro* to septic plasma, resulting in significant increase in levels of abundance of ACSL1 transcripts. Therefore, the most relevant topics selected for the assessment of potential gaps in biomedical knowledge were “sepsis” and “neutrophils.”

One of the 625 articles constituting the ACSL1 literature contained the keywords “sepsis,” “septic,” “septicemia,” or “septiceamia,” This work examined responses to endotoxin treatment in mice and made reference to sepsis for context without actually studying septic animals (17). The authors report a decrease in ACSL1 expression in liver and adipose tissues in mice treated by LPS. Three articles contained the keywords “neutrophil” or “neutrophils” in its title or abstract. These reports, which were all published in the early 1990s, employed an ACSL1 inhibitor, triacsin C, to investigate its role in regulating production of various factors, including superoxide anions, which mediate in part neutrophils microbicidal activity, or eicosanoids, which are oxidized fatty acids possessing pleiotropic signaling properties (18–20).

It was thus possible to conclude at this stage:

- First that the increase in ACSL1 abundance observed in neutrophils exposed *in vitro* to septic serum is a relevant phenomenon *in vivo*, since it was also increased in the blood of septic patients.
- Second, that this phenomenon can be considered to be robust since it was observed in multiple studies across various clinical and study settings.
- Third, that an increase in ACSL1 expression has not been reported in the literature in the context of sepsis to date. And that a role for ACSL1 in neutrophil immunobiology has been described, but not in the context of sepsis. Furthermore, it is an area of research which has been inactive for at least the past two decades.

The next steps consisted in bringing additional context via a general review of the literature retrieved for ACSL1 and four other members of the long chain acyl-CoA synthetase family. Several reference transcriptome datasets which are available publicly were also consulted in order to complete this picture.

## Review of ACSL Family Members Function and Role in Disease

In order to draw inferences on the possible role played by ACSL1 in sepsis, context was obtained by conducting a background literature review. As a first step, each of the workshop participants were tasked to retrieve literature for one of the five known members of the family of human ACSL genes. Once they had optimized PubMed queries designed to retrieve the literature of each respective family members, keywords identified “manually” in titles of articles were recorded and categorized (categories included for instance: disease, tissue, cell type, biomolecule, biological process). Recording the frequency at which those keywords occurred in the literature allowed in turn the identification of the main research themes for each family member.

Furthermore, tissue-specific expression of ACSL family members was looked up in BioGPS, a publicly accessible data browsing portal which comprises several reference transcriptome datasets (21). Relative abundance of ACSL family members in human tissues is shown in Supplementary Figure 1 and discussed below.

### ACSL1

Main themes for ACSL1 identified via profiling of its literature were: *lipid metabolism, fatty acid uptake, insulin, and muscle*. ACSL1 is the main ACSL family member expressed in the liver (22). Substrate specificity is highest for saturated and monounsaturated FA that are 16–18 carbons in length (23). Overexpression of ACSL1 in rat hepatocytes increased oleate incorporation into diacylglycerol and phospholipids (24). Liver-specific knockout reduced TAG synthesis and FA oxidation (22). ACSL1 expression has also been described in skeletal muscle (25), prostate (26), and adipose tissue (27). In mice, ACSL1 was specifically required for FA beta-oxidation in both muscle and adipose tissue (25, 27). In human adipocytes, ACSL1 expression has been associated with increased lipid content and insulin sensitivity (28). Additionally, ACSL1 knockdown in adipocytes resulted in upregulation of proinflammatory chemokine (C-C motif) ligand 2 (*CCL2*) and chemokine (C-C motif) ligand 5 (*CCL5*) and the macrophage associated surface antigen cluster of differentiation 14 (*CD14*) (28). Although ACSL1 was found to localize to mitochondria, a role for FA uptake in adipocytes has been proposed by the mechanism of metabolic trapping (29). A similar role has been described in human hepatoma cells (30). Upregulation of ACSL1 has been observed in various cancer types, including breast cancer (31), colon cancer (32), and liver cancer (30, 33, 34). Interestingly, aspirin down-regulates ACSL1 expression in liver cancer cells and suppresses abnormal lipid metabolism via inhibition of NFκB-ACSL1 signaling (34). Consistently with reports from the literature, BioGPS profiles indicated predominant expression of ACSL1 in liver and prostate tissue (22, 26) (Supplementary Figure 1). Interestingly, it is also by far the member of the ACSL family that is most highly expressed in blood, which is another indication of the more prominent role ACSL1 might play in immunity in comparison with other ACSLs.

### ACSL3

Main themes for ACSL3 identified via profiling of its literature were: *lipid droplet, liver, and goose fatty liver*. Expression of ACSL3 has been described in various tissues including the brain (35), pancreatic islet cells (36), upper small intestine (37), vascular smooth muscle cells (38), liver (39–41), and fetal liver (42). Transcriptome data available via the BioGPS portal and depicted in Supplementary Figure 1 shows ACSL3 as the member of the family with the highest degree of restriction to the CNS. ACSL3 is typically found on cellular lipid droplets and the endoplasmic reticulum (ER) (43). Different functions of ACSL3 have been reported across these different tissue types. As ACSL3 moves from the ER to newly formed lipid droplets upon exogenous FA treatment, a role for lipid synthesis has been proposed (44). In pancreatic cells, ACSL3 localized to insulin secretory granules and ACSL3 knockdown inhibited glucose-stimulated insulin release (36). In vascular smooth muscle cells, NFκB-ACSL3 signaling was identified to mediate palmitic acid induced osteoblastic differentiation (38). In the brain, activation of long-chain FA by ACSL is required for FA incorporation in signaling molecules as well as structural components, such as myelin (45).

### ACSL4

Main themes for ACSL4 identified via profiling of its literature were: *fatty acid metabolism and arachidonic acid metabolism/regulation*. ACSL4 gene expression has mostly been reported in the adrenal gland, liver, and adipose tissue (1, 46, 47). This is also consistent with the transcriptional profiling data available via the BioGPS portal, which shows a predominance of ACSL4 expression in fetal liver (Supplementary Figure 1). Overexpression of this gene has previously been reported in fetal liver in the Human Integrated Protein Expression Database (48). However, a specific role for ACSL4 in fetal liver or liver development has not been described. In adult liver ACSL1 is the predominant form of long-chain acyl-CoA synthetase. The substrate preference of ACSL4 is arachidonic acid (AA) and eicosapentanoic acid (46). Next to a role for in AA metabolism (46, 49–51), ACSL4 is involved in intracellular lipid storage (52), and cholesterol transport from ER to the mitochondria (53). A splice variant of ACSL4 was found in ER tubules of hippocampal neurons in rats, and ACSL4 knockdown resulted in reduction in dendritic spine density (54). In humans, ACSL4 has been associated with X-linked mental retardation (55).

### ACSL5

Main themes for ACSL5 identified via profiling of its literature were: *fatty acid uptake, mitochondrial metabolism, and physiology of the intestine*. ACSL5 is most abundantly expressed in the small intestine, according to literature reports (1, 56–59) and bioGPS ACSL5 expression profiles. Abundance increases along the crypt-villus axis and is assumed to promote apoptosis in enterocytes at the villus tip (60). Additionally, an anabolic function of ACSL5 has been proposed, supporting FA uptake by cells (39, 61). Interestingly, ACSL5 was found to be associated with decreased Wnt signaling in normal human intestinal tissues. Palmitolylation of Wnt2B mediated by ACSL5 results in accumulation of this protein to mitochondria and consequently decreased Wnt activity (58). This finding illustrates that next to regulation of lipid metabolism, ACSL family members can also impact intracellular pathways by protein-lipid modifications. Low expression of ACSL5 in colorectal cancer has been associated with increased risk of tumor recurrence (62).

### ACSL6

Main themes for ACSL6 identified via profiling of its literature were: *fatty acid, DHA, and schizophrenia*. Literature reports ACSL6 to be almost exclusively expressed in the brain (61, 63–65). This tissue restricted expression pattern suggests ACSL6 has a specific role in regulation of brain lipid metabolism. ACSL6 was found to be required for enrichment of omega-3 FA Docosahexaenoic acid that confers protection against numerous neurological diseases (64). Correspondingly, genetic polymorphism of the *ACSL6* gene have previously been associated with schizophrenia (66, 67). Reference transcriptome data available via the BioGPS portal confirms restriction of ACSL6 expression to the CNS, with highest levels observed in the pineal gland, hypothalamus and prefrontal cortex. However, expression levels are much lower than that of ACSL3 which minimizes ACSL6 contribution when considering overall expression of ACSL family members across multiple tissues (Supplementary Figure 1).

## A Review of the Literature on the Role of ACSL Family Members in Immunity

Next focus of the literature and public transcriptome datasets review shifted to examining the possible role of ACSL family members in immunity in general, and more particularly in sepsis and neutrophil immunobiology.

### Monocytes and Macrophages

Whereas the regulation of ACSL family members has been relatively well-described in solid organs, only a small number of studies have examined the role of ACSLs in circulating leukocytes. Compared to other immune cell types, ACSL family members have been most frequently related to monocytes and macrophages. This is consistent with a function of macrophages in lipid metabolism that is mostly observed in the lung and liver (68). Macrophages ingest LDL, VLDL, and oxidized lipoproteins and digest them in the lysosome (69). Excess cholesterol is exported from the cell to HDL, and free FA will be either oxidized to generate energy or used for FA synthesis (68). The requirement of FA oxidation or FA synthesis is dependent of the macrophage's phenotype (e.g., proinflammatory M1 or anti-inflammatory M2) (70, 71). In fact, interfering with macrophage FA oxidation induces phenotypical changes, suggesting that lipid metabolism may control macrophage phenotype (72–74).

Inhibition of ACSL1, ACSL3, and ACSL4 by triacsin C, significantly attenuated FA incorporation into diglyceride, triglyceride, and phospholipids in human monocyte-derived macrophages (75). ACSL4-specific inhibition by rosiglitazone showed that this member has restricted effect on FA partitioning to phospholipids, suggesting individual family members have distinct functions (75). Similarly, triacsin C inhibited cytosolic lipid droplet formation in mouse macrophages (76). Treatment of mouse peritoneal macrophages with free FAs in the presence of triacsin C reduced triglyceride synthesis, but increased intracellular free FA levels, hereby inducing lipotoxicity (77). In mouse macrophages, ACSL1 overexpression resulted in reduced levels of the reverse cholesterol transporter ABCA1, suggesting a role in regulating cholesterol efflux (78).

Upregulation of ACSL1 has been observed in response to various stimuli and in diverse pathological conditions. Human PBMC-derived macrophages infected with mycobacterium tuberculosis had an increased expression of ACSL1 and increased lipid droplet formation (79). In patients with type 1 diabetes, ACSL expression was increased in monocytes and macrophages (80). Gram-negative bacteria and LPS induce expression of ACSL1 which correlated with increased phospholipids turnover (81). Toll like receptor agonists were also found to promote prolonged triglyceride retention that associated with increases in ACSL1 (82, 83). Tyrosine kinase, an integral component of the JAK-STAT pathway was found to affect protein expression of ACSL4 in bone marrow-derived macrophages from mice (84).

Abundance of ACSL1 proteins has been found to be increased in monocytes and macrophages displaying inflammatory phenotypes (80, 85). ACSL1-deficient macrophages in a mouse model of diabetes, showed a marked reduction in arachidonoyl-CoA levels. In these macrophages, beta-oxidation and lipid accumulation were not impaired, suggesting a specific role in arachidonoyl handling (86). ACSL1 expression in macrophages associated with an inflammatory phenotype characterized by upregulation of cytokines IL-1b, IL-6, and CCL2, most likely mediated by levels of arachidonoyl-CoA that is required for eicosanoid production (80, 86). Another study also demonstrated that ACSL1 expression effects inflammatory activity of cultured human THP-1 macrophages. Both ACSL1 inhibition and knockdown by siRNA suppressed palmitate-induced TNF-α expression. Eicosapentaenoic acid was shown to have an anti-inflammatory effect through inhibition of ACSL1 expression (87).

Only few articles describe the potential roles for ACSL in neutrophils. Evidence for the effects of ACSLs in neutrophils dates back to the early 1990s and is derived from experiments treating neutrophils with triacsin C, an ACSL inhibitor. In rat neutrophils stimulated with A23187 or fMLP, pretreatment with triacsin C upregulated platelet-activating factor. Inhibition of ACSL resulted in inhibition of acylation of lyso-phospholipids, enhancing in turn the supply of substrate for PAF synthesis (18). The same group also found that ACSL is involved in the acylation of arachidonic acid in rat neutrophils. Pretreatment with triacsin C inhibited incorporation of arachidonic acid into the cells and resulted to an increased release of arachidonic acid metabolites (19). In human neutrophils, a more direct relation between ACSL activity and neutrophil function was found. Triacsin C inhibited generation of superoxide anion and release of azurophilic granules by human neutrophils. As reactive oxygen species (ROS), such as superoxide anion play an important role in neutrophils protective mechanisms, these findings suggest that ACSL activity is important for neutrophil functions (88). A proposed reason for the requirement of acyl-CoA in both neutrophil respiratory burst and the modulation of neutrophil degranulation is that both processes would require phospholipid synthesis (88, 89). In fact, more recently, a key role for phospholipids has been described in increasing and dampening of the production of ROS during the oxidative burst (90).

### Other Immune Cell Types

For other immune cell subsets, there is very limited evidence for the function of ACSL. In NK-cells and B-cells, no articles were found for either ACSL, and only two articles describe ACSL in T-cells. An early study in murine thymoma and cytotoxic T lymphocyte clones pointed to the presence of two distinct ACSL family members in T-cells. The enzyme described in this study and that is currently known as ACSL4 showed activity selective for arachidonic acid (87). The second enzyme displayed broad substrate specificity (87), implying expression of an additional ACSL member in T cells (91). A few decades later, ACSL5 expression was reported to be higher in PBMCs of patients with systemic lupus erythematosus compared to healthy controls. In cultured Jurkat T-cells, ACSL5 expression was increased during induced apoptosis, while siRNA downregulation of ACSL5 decreased induced apoptosis (92). These findings suggest a potential role for ACSL5 in T-cell apoptosis.

## A Review of Public Reference Transcriptome Datasets

### Restriction of Expression of ACSL Family Members in Blood Leukocytes

Additional context was obtained through a review of available public reference datasets. A first dataset was accessed to verify the restriction of ACSL family members across different blood leukocyte populations. The dataset with ID GSE60424 deposited in GEO by Linsley et al. comprises RNAseq profiles of six human immune cell subsets isolated from healthy subjects, as well as subjects with multiple sclerosis, ALS, Type 1 Diabetes or sepsis (*N* = 19) (93). ACSL expression profiles for this dataset were accessed using the GXB web application that permits interactive browsing of gene expression data (94). Various dataset that are available in NCBI's Gene Expression Omnibus (GEO) have been made accessible using this tool (95–99).

**Figure 3:**
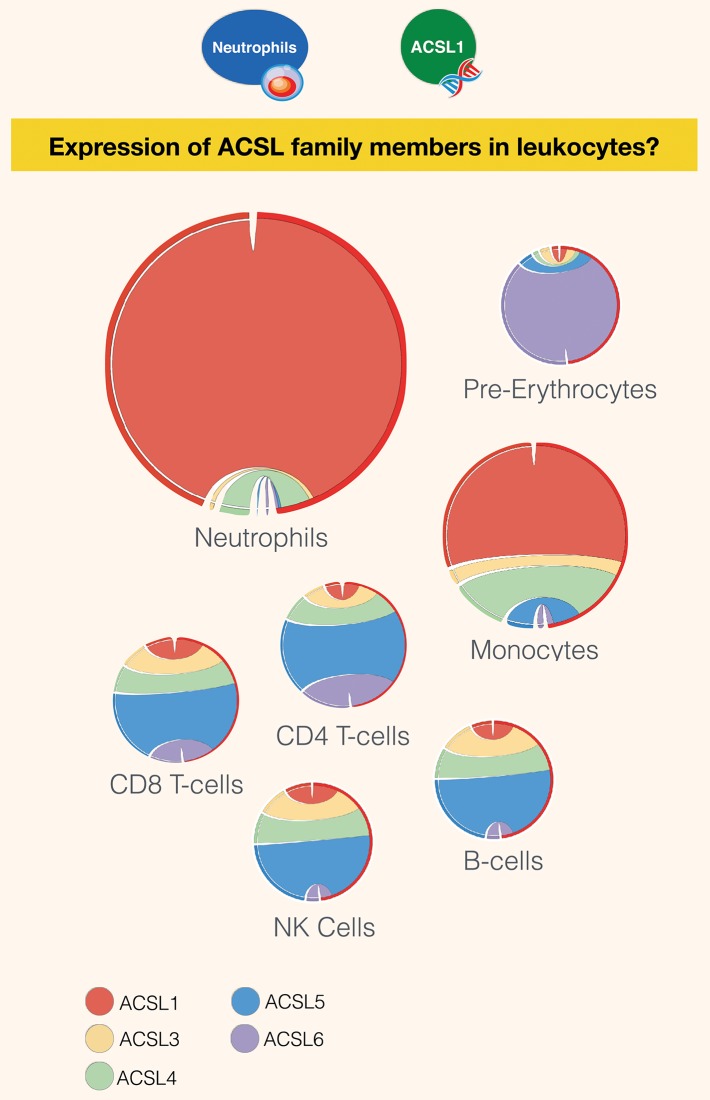
Restriction of expression of ACSL family members transcripts in human leukocyte populations. Relative transcript abundance levels for the 5 members of the ACSL family are represented with cord plots for 7 different blood leukocyte populations. Expression data used to generate the plots were obtained from public domain RNAseq dataset with accession ID GSE60424 (93), with the exception pre-erythrocytes expression data, which was obtained from the BioGPS dataset (21). Each family member is represented by a different color: ACSL1 (red), ACSL2 (beige), ACSL4 (green), ACSL5 (blue), and ACSL6 (purple). The size of the circle is proportional to relative expression levels of ACSLs. For instance, expression in neutrophils was about four times higher than in monocytes and ten times higher than in other cell populations.

Interestingly, despite the limited number of publications linking the activity of the long chain acyl-CoA synthetases to immunity, members of the family were found to be robustly expressed across various immune cell subsets (Figure 3). While ACSL5 is the most highly expressed in CD8 and CD4 T-cells, NK cells, and B-cells [GXB link (100)], neutrophils and monocytes were found to mostly express ACSL1 [GXB link (101)]. The most abundant family member in pre-erythrocytes is ACSL6. Neutrophils have the overall highest expression of ACSL, followed by monocytes, as represented by the size of the circle diagrams in Figure 3. The relative expression profile of ACSLs in monocytes is consistent with the identified articles using our literature search for monocytes and macrophages. However, a remarkably low number of articles describe the role of ACSL in neutrophils, which is indicative of the presence of a current gap in biomedical knowledge that remains to be filled.

### Regulation of ACSL1 and ACSL4 in Blood Cultivated With a Wide Range of Immune Stimuli

**Figure 4:**
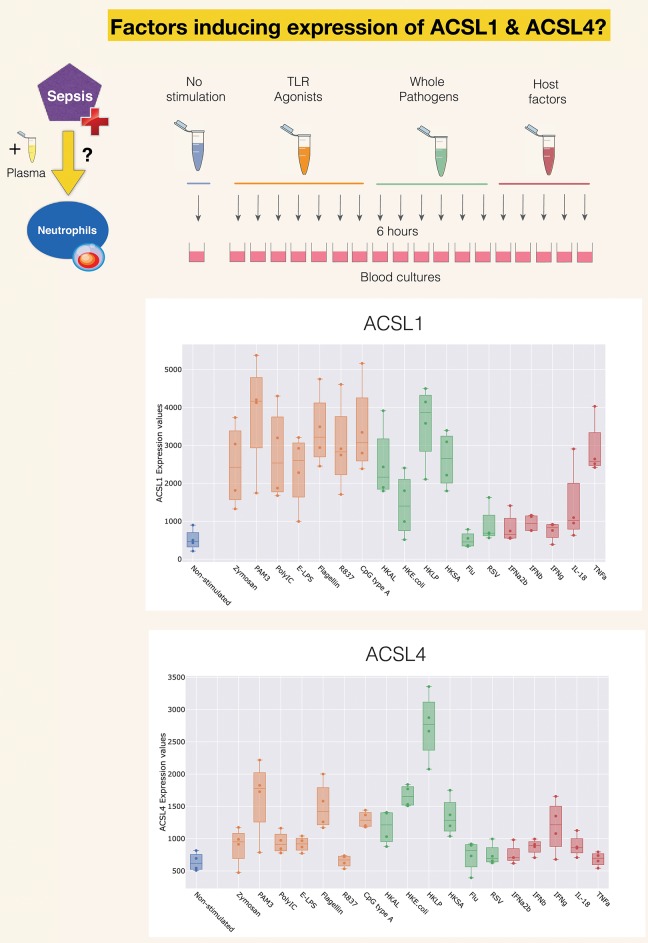
Induction of ACSL1 and ACSL4 expression in response to immune stimulation. The data presented here was generated by Obermoser et al. (GSE30101) (102). The box plots show levels of abundance of ACSL1 and ACSL4 in whole blood of four healthy subjects incubated for 6 h. at 37°C with 18 different immune stimuli or left unstimulated. Conditions included Toll-like receptors agonists PAM3, Zymosan, Poly IC, E-LPS, Flagellin, R837, CpG Type A, heat-killed *Escherichia coli* (HK *E. coli*), heat-killed *Legionella pneumophila* (HKLP), heat-killed *Acholeplasma laidlawii* (HKAL), and heat-killed *Staphylococcus aureus* (HKSA); live influenza A virus (Flu) and live respiratory syncytial virus (RSV), and the host-derived immunostimulatory molecules IL18, TNF-α, IFN-α2b, IFN-β, IFN-γ.

A second reference dataset was accessed, which permits to assess changes in levels of expression of ACSLs in blood in response to a wide range of pathogen-derived and host-derived immune stimuli. This dataset was contributed to GEO by Obermoser et al. [GSE30101 (102)]. Overall, abundance of most ACSL family members was found to be increased, except for ACSL6. The highest rates of increase were observed for ACSL1, ACSL4, as shown Figure 4. Patterns of induction were strikingly different for those two ACSLs, with ACSL1 showing increases in response to most purified pathogen derived molecules and heat-killed bacteria, as well as the proinflammatory factor TNF (103). ACSL4 induction was more narrowly restricted, showing upregulation mostly in response to heat-killed bacteria and purified components, such as PAM3 (TLR1/TLR2 ligand), and flagellin (TLR5 ligand) (104). Notably, heat-killed *Legionella pneumophila*, a flagellated gram negative bacteria, was the most potent inducer of ACSL4 expression in this culture system. PAM3 [Pam (3) CSK (4)] is a strong stimulator of TLR5 expression in neutrophils (105). Not shown on this figure but accessible via the GXB are profiles of ACSL3 and ACSL5, which interestingly in the case of ACSL3 is clearly restricted to stimulation via Poly:IC (TLR ligand) and beta interferon (106). And in the case of ACSL5 pattern of induction were to some extent similar to those observed for ACSL4 (heat killed bacteria + PAM3 + Flagellin) (107).

## Inferring the Role of ACSL1 in Neutrophils in the Context of Sepsis

Sepsis presents as a spectrum of systemic manifestations resulting from a pathogenic infectious process. Overt inflammation and activation of the innate immune system is often mentioned as one of the hallmarks of sepsis. But this response is also accompanied by profound changes in metabolism homeostasis and followed at later stages by potent immunosuppression, marked reduction in inflammation and generally promotion of a milieu favorable to tissue repair (108). During the early stages of sepsis neutrophils play an important role in promoting microbial clearance through microbicidal and pro-inflammatory effector function. But they may present during the resolution phase of the disease with altered cellular phenotype ranging from increased lifespan, impaired migration to site of infection, and decreased antimicrobial function (109–111).

As shown above the abundance of ACSL1 transcripts is increased in sepsis in a wide range of study settings, but literature describing mechanisms regulating its expression or its role in this context is lacking.

The review of the literature and of public transcriptional profiling data that we have conducted provides some clues with regards to these aspects of the biology of ACSL1.

### Regulation of ACSL1 Expression

**Figure 5:**
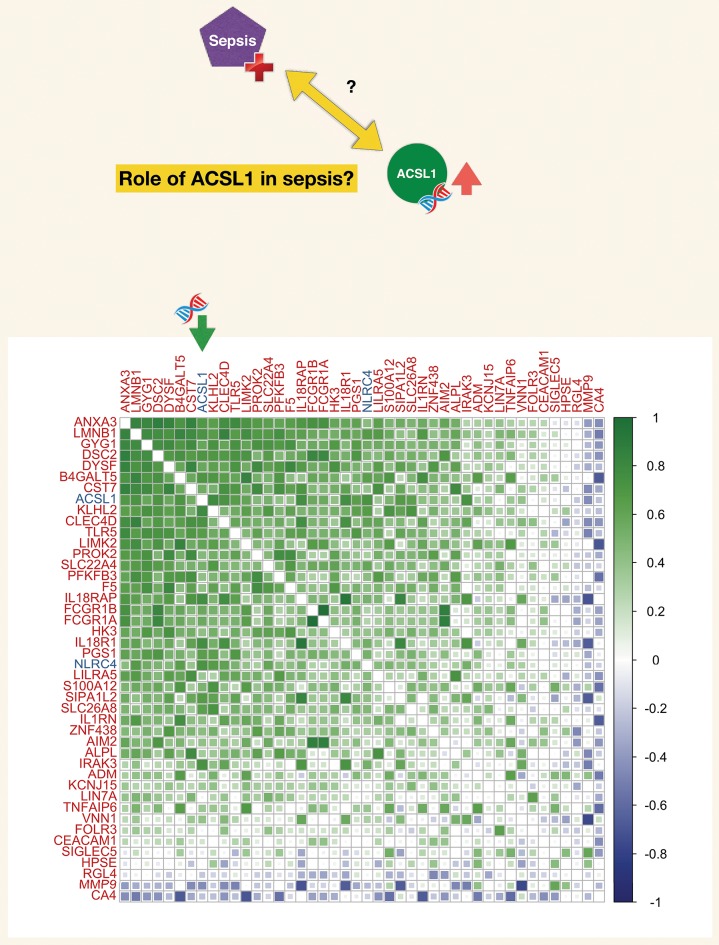
Correlation matrix for a set of genes constituting a blood transcriptional module associated functionally with neutrophil-driven inflammation (M13.12). The blood transcriptional module is part of a recently described repertoire constructed on the basis of co-expression across a 16 different immune/pathological states (described in the text). The list of genes constituting this module (M13.12) comprised ACSL1. The matrix represents degree of correlation calculated for each gene pair for the septic plasma neutrophil exposure dataset.

The increase in abundance of ACSL1 transcripts observed in whole blood of septic patients may be attributed on one hand to the upregulation of its expression *in vivo*, but on the other hand it may also be the result of an increase in the relative abundance in blood of cellular populations preferentially expressing ACSL1. In addition, it could of course also be the result of the combination of both factors. The fact that an increase in ACSL1 abundance was also observed after 6-h stimulation *in vitro* in blood cultures and in neutrophil cultures suggests that changes in ACSL1 abundance are likely to be at least in part attributable to regulation of gene expression. In these *in vitro* blood cultures levels of expression of ACSL1 were increased in response to whole bacteria or purified pathogen-derived molecules as well as host-derived inflammatory factors, such as TNF (Figure 5). Both pathogen- and host-derived immunostimulatory molecules may be present in septic plasma and contribute to the induction of ACSL1 and ACSL4 expression observed in neutrophils cultures (Figure 1), as well as in whole blood *in vivo* (Figure 2). Of potential interest is the apparent differences in patterns of induction of ACSL1 and ACSL4, with as noted above a preferential induction of ACSL4 by flagellin and the heat killed flagellated bacterium *Legionella pneumophila*.

Another take on a potential mechanisms regulating ACSL1 expression originates from the fact that lipid metabolism is frequently deregulated during sepsis, characterized by changes in cholesterol, HDL and apolipoproteins in plasma of septic patients (112–114). Evidence from an *in vitro* study suggests that exposure to fatty acids significantly increases neutrophil adherence and release of specific granules (115). Therefore, it is possible that the changes in lipid content in plasma of septic patients could be responsible for the observed increased expression of ACSL1 in neutrophils, and consequently, altered neutrophil function.

### A Potential Role for ACSL1 in Sepsis?

The actual role played by ACSL1 in sepsis and its contribution to the pathophysiology of this condition remains to be defined. In the meantime, some inferences can be drawn on the basis of current knowledge of the role of ACSL1 in neutrophil and monocyte/macrophage immunobiology on one hand and on omics data co-expression analyses on the other hand.

#### Inferences Based on the Literature

As has been described in more details above, most of what is currently known of the role of ACSL1 in neutrophils is based on three articles published in the early 1990s (18–20). These employed the inhibitor triacsin C to demonstrate ACSL1 involvement in promoting production of superoxide anions and degranulation, as well as decreasing production of ecoisanoid and platelet activation factor. In the context of sepsis production of reactive oxygen species by neutrophils accounts in part for their microbicidal activity and contributes to pathogen clearance (110). However, when oxidative stress levels increase past a critical threshold cell apoptosis and end organ damage may ensue.

A recent study in monocytes, which also employed triacsin C, demonstrated a role for ACSL1 in mediating inflammatory activity and phenotype following TNF stimulation (85). Another very recent report was published demonstrating ACSL1 role in driving production of IL1B in macrophages via regulation of fatty acid metabolism in mitochondria (116). The same study shows that ACSL1 expression is upregulated following engagement of TLR4 in macrophages and, furthermore, that knockdown of ACSL1 in macrophages results in decreased NLRP3 inflammasome activation. Altogether this work points to a pro-inflammatory role for ACSL1 in monocyte/macrophages, possibly underlying inflammasome activation observed in metabolic disorders characterized by high levels of circulating saturated fatty acids. Data gathered from public repositories presented in this review suggest that neutrophils may play a greater role than previously recognized in this process.

#### Inferences Based on Co-expression Analysis

Co-expression analyses are a common means by which putative function may be assigned to genes (referred to as “guilt by association”). Here we took advantage of a repertoire of transcriptional modules developed by our team on the basis of co-expression analysis of whole blood transcriptome profiles obtained for 16 disease states across nearly 1000 subjects. Such repertoires of co-expressed gene sets have been in use for over 10 years (102, 117), and the latest iteration used in this case is described in a manuscript deposited on the BioRxiv pre-print server (118). ACSL1 was included among a set of 55 transcripts which were assigned the module identifier M12.13 (one of a total of 381 modules). Functional profiling of this gene set was performed via: (1) gene ontology enrichment, (2) Pathway analysis (Ingenuity pathway analysis, Qiagen), (3) Literature profiling (Literature lab, acumenta). In addition expression levels were mapped for two different reference datasets: (1) Blood leukocyte RNAseq dataset [GSE60424 (93)] and (2) Hematopoeitic progenitor reference dataset [GSE24759 (119)]. Ontology, pathway and literature keyword enrichment all point to a core group of genes constituting this module being strongly associated with inflammation and in some cases more specifically inflammasomes. Examples of genes most prominently associated with inflammation, NOD-Like Receptor and Toll-like receptor pathways include IRAK3, TLR5, IL18R1, IL18RAP, IL1RN, as well as the inflammasomes molecules AIM2 and NLRC4. Other annotations mention phagocytes and myeloid cells as well as host-pathogen interaction. This fits well with leukocyte transcript restriction data accessed for this set of 55 genes it shows a markedly higher expression in neutrophils. Levels of expression of those transcripts were also elevated in monocytes in the context of sepsis (Supplementary Figure 2). More details along with functional analysis reports for module M13.12 is available via an interactive presentation (https://prezi.com/view/pGdJTaNfTCaiJzJvkwif/). A high degree of correlation was also observed among the transcripts forming M13.12 in the septic plasma neutrophil exposure dataset used as a starting point for this work (Figure 5). Correlation coefficient for ACSL1 and NLRC4 for this dataset was 0.72.

Correlations were also measured for two “canonical” inflammasome and fatty acid metabolism gene set (identified via a knowledge-driven approach for the purpose of offering targeted “RT2 profiler PCR arrays”). As shown on Supplementary Figure 3 strong positive correlations were observed between ACSL1 and nearly half of the transcripts that belong to the inflammasome panel (including most notably NLRC4, but also NLRP3, CASP1, CASP5, TXNIP). In the fatty acid metabolism panel the strongest correlation observed was with ACSL4, followed by PPA1, a member of the inorganic pyrophosphatase family, and ACOT7, an Acyl-CoA thioesterase.

Taken together, inferences that could be drawn based on both profiling of the literature and transcript co-expression analysis confirm a likely role of ACSL1 in driving inflammasome-mediated release of pro-inflammatory factors by neutrophils during sepsis.

### Clinical/Translational Implications

**Figure 6:**
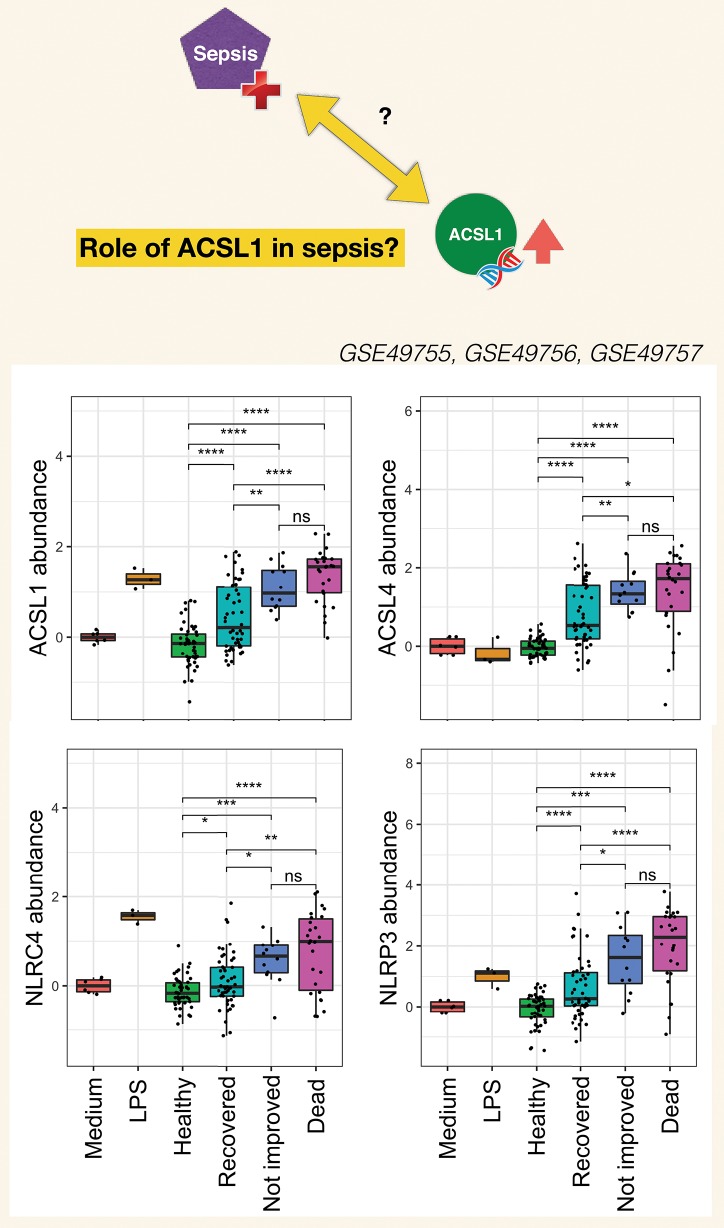
Clinical significance of increases in ACSL1, ACSL4, and Inflammasome genes in the septic plasma neutrophil exposure dataset. For this figure all three datasets from this dataset series published as part of the work of Khaenam et al. were combined (see text for details). Fold changes over average of cells cultured with medium only are shown. Available clinical information was used to assign sepsis subjects to either one of three groups: Recovered, Not improved and Dead. Significance between the different groups is indicated above each graph with *t*-test ^*^p < 0.05, ^**^p < 0.01, ^***^p < 0.001, ^****^p < 0.0001.

The burden of sepsis remains high, with poor outcomes observed especially in developing countries. Despite several attempts at developing drugs that would control overt inflammation no immune-modifying treatment is currently available for the management of sepsis (120). Contributing to a better understanding of mechanisms underlying inflammatory responses at play during sepsis should permit to refine targeting strategies. Pinpointing the potential involvement of ACSL1 in this process may thus prove of value. Choosing the timing of such therapeutic intervention is particularly difficult given the dynamic nature of the immune response during sepsis which is characterized by an initial “cytokine storm” followed by a phase during which the immune system becomes hyporesponsive. The development of means to monitor the development of the inflammatory response and guide treatment decision is another gap worth addressing. It is however difficult to find adequate longitudinal data that would permit to address this point. Generating such data could be another worthwhile endeavor, especially since levels of induction of ACSL1 following neutrophil exposure to septic plasma were significantly higher in response to plasma of patients who died from sepsis or did not initially improve than in response to plasma of patients who recovered (Figure 6). Similar differences were found for ACSL4, NLRC4, and NLRP3.

### Working Model and Follow on Investigations

**Figure 7:**
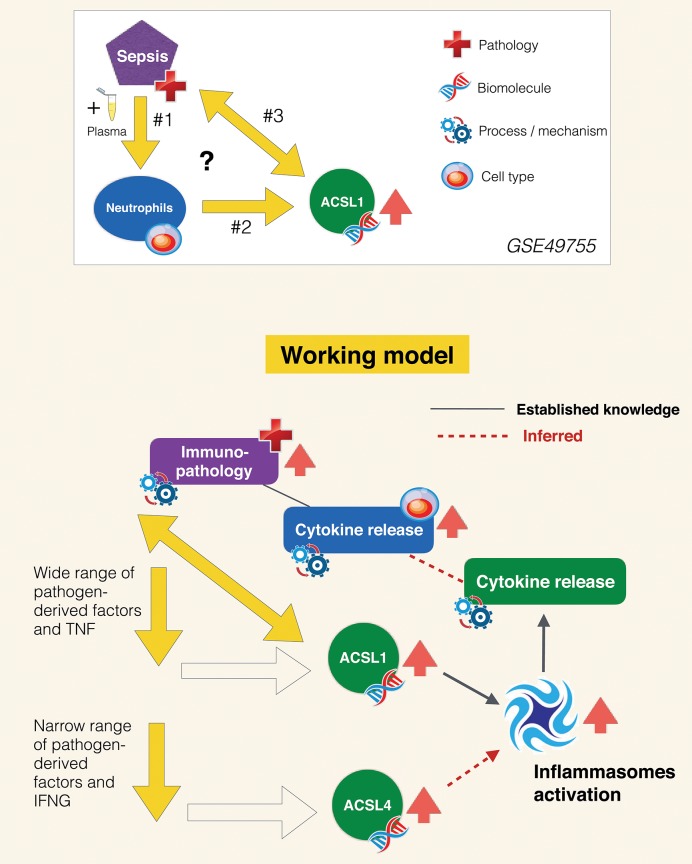
Inferred role of ACSL1 in sepsis. The experimental context in which the increase in ACSL1 expression was initially observed is represented in the panel above (GSE49755). Open questions include: #1 What are the factors in the plasma of septic patients that induce ACSL1 expression in neutrophils? #2 Which signaling pathway is involved in regulation of ACSL1 following plasma exposure? #3 What is the role of ACSL1 in sepsis? Regarding question #1 (arrows down): briefly as suggested by the data presented on Figure 4 ACSL1 is induced in blood by a wide range of pathogen-derived factors and ACSL4 by a narrower range of factors (putatively attributed to the flagellated bacteria TLR5 agonist, flagellin). Regarding question #2 (oblique arrow): ACSL1 is known to contribute to the activation of inflammasomes in macrophages, which in turn leads to release of proinflammatory factors (116). Based on differential and co-expression analyses we infer that it might also be the case of ACSL4, for instance during responses to infection caused by flagellated bacteria. We also infer that ACSL-mediated inflammasome activation operates in neutrophils and also in the context of sepsis, which are also points that remain to be established. Neutrophil contribution to the cytokine storm observed during sepsis and subsequent immunopathology is well-documented. We thus assume that upregulation of ACSL1 observed during sepsis is of potential clinical relevance. An assertion that is supported by differential levels of induction observed following exposure to plasma of subject who recovered vs. those who died (Figure 6).

As stated in the introduction, the primary goals of this joint review of the literature and public transcript profiling data were first to explore a potential gap in biomedical knowledge and second to obtain insights that may serve to guide future experimentation. Inferences drawn from literature and transcriptome data reviews are summarized in Figure 7. A role has been very recently attributed to ACSL1 in inflammasome activation and proinflammatory cytokine release in macrophages (116). This was described by the authors as being mediated via the formation of intracellular fatty acid crystals which are causing lysosomal damage and in turn inflammasome activation and IL1B release. But the fact that this pathway may operate in neutrophils and in the context of sepsis has not yet been recognized and it is something that co-expression analyses presented in Figure 5 and Supplementary Figure 3 strongly support, and so does restriction of its expression among blood leukocyte populations (Figure 3). Notably increase in abundance were observed *in vitro* in response to septic serum (Figure 1), but also *in vivo* in whole blood or neutrophil of septic patients (Figure 2, Supplementary Figure 2). The transcriptome profiling data that we have examined also suggest an involvement of ACSL4 in activation of inflammasomes in neutrophils, but possibly less ubiquitously than ACSL1, as suggested by narrower range of response to pathogen-derived activators (Figure 4). Figure 7 summarizes the points that have been inferred with regards to regulation of ACSL1 and ACSL4 expression in leukocytes and their involvement in sepsis pathogenesis. Figure 6 provides a first indication of the potential clinical relevance of the phenomenon being described.

Follow on work should permit to characterize more precisely the role of ACSL1 in neutrophils and sepsis. Gene silencing or knock down technologies, such as siRNA or Crispr/Cas9 could for instance address the lack of specificity of triacsin C (since it also inhibits other ACSL family members, including ACSL4). The panel of stimuli employed in the experiment described in Figure 4 could be employed in order to screen for pathway specific alterations/defects. General activation and in particular production of reactive oxygen species could be primarily be used as readout. But use of omics scale profiling approaches would also permit to obtain a more global perspective on the range of effects ACSL1 may have on neutrophil function. In order to investigate the role of ACSL1 *in vivo* an experiment would consist in monitoring survival of Acsl1^−/−^ mice [derived and employed earlier in a different context (22, 27)] in a cecal ligation sepsis model or following endotoxin challenge. Finally, it may also be interesting to explore the possibility of ACSL1 and ACSL4 being involved in responses to distinct bacterial species, as suggested by the flagellin/flagellated bacteria bias observed in the ACSL4 response shown in Figure 4.

## Author Contributions

JR, MG, DR, AJ, DB, and DC: conceptualization. MG, MT, and DR: data curation and validation. JR, DR, and DC: visualization. JR, MG, EH, YM, PS, and DC: analysis and interpretation. JR and DC: writing of the first draft. SB, DB, and DC: funding acquisition. MG, MT, DR, and DC: methodology development. MA, MT, and SB: software development and database maintenance. JR, MG, EH, YM, PS, MT, MA, WH, SB, DR, AJ, DB, and DC: writing–review and editing. The contributor's roles listed above follow the Contributor Roles Taxonomy (CRediT) managed by The Consortia Advancing Standards in Research Administration Information (CASRAI) (https://casrai.org/credit/).

### Conflict of Interest

The authors declare that the research was conducted in the absence of any commercial or financial relationships that could be construed as a potential conflict of interest.

## Supplementary Material

Supplementary Figure 1 Tissue restriction of ACSL family members. Relative abundance levels of the 5 members of the ACSL family across 8 different human tissues is shown is this figure. Expression data used to generate the plots were obtained from the BioGPS dataset (21). Each family member is represented by a different color: ACSL1 (red), ACSL2 (beige), ACSL4 (green), ACSL5 (blue), and ACSL6 (purple). Predominance of a given color in a circle denotes predominance of expression of the corresponding ACSL family member in the tissue in question. Cord plots were generated using the circlize R package (121).

Supplementary Figure 2 Abundance levels of a co-expressed geneset comprising ACSL1 across leukocyte populations and disease states. A reference public domain dataset was downloaded from GEO (GSE60424) in which RNAseq profiles were generated for 6 leukocyte subsets (legend: cell types) isolated from 20 blood samples collected from healthy subjects and subjects with a wide range of pathologies (legend: disease). Z-scores were computed from normalized counts and displayed on a heatmap (blue/gray = low expression; yellow = high expression. Samples were arranged according to grouping information and genes were arranged based on similarities in patterns of expression across samples.

Supplementary Figure 3 Correlation matrix for “inflammasome” and “fatty acid metabolism” gene sets. Lists of genes known to be associated with inflammasome and fatty acid metabolism were derived from targeted RT-PCR panels (referenced in the text). ACSL1 was added to the inflammasome list. The matrix represents degree of correlation calculated for each gene pair using the septic plasma neutrophil exposure dataset (GSE49755).
