# Supplementary figures and images for: Long-Chain Acyl-CoA Synthetase 1 Role in Sepsis and Immunity: Perspectives From a Parallel Review of Public Transcriptome Datasets and of the Literature
Source: Front Immunol. 2019 Oct 18;10:2410. doi: 10.3389/fimmu.2019.02410 (PMC6813721; doi:10.3389/fimmu.2019.02410)

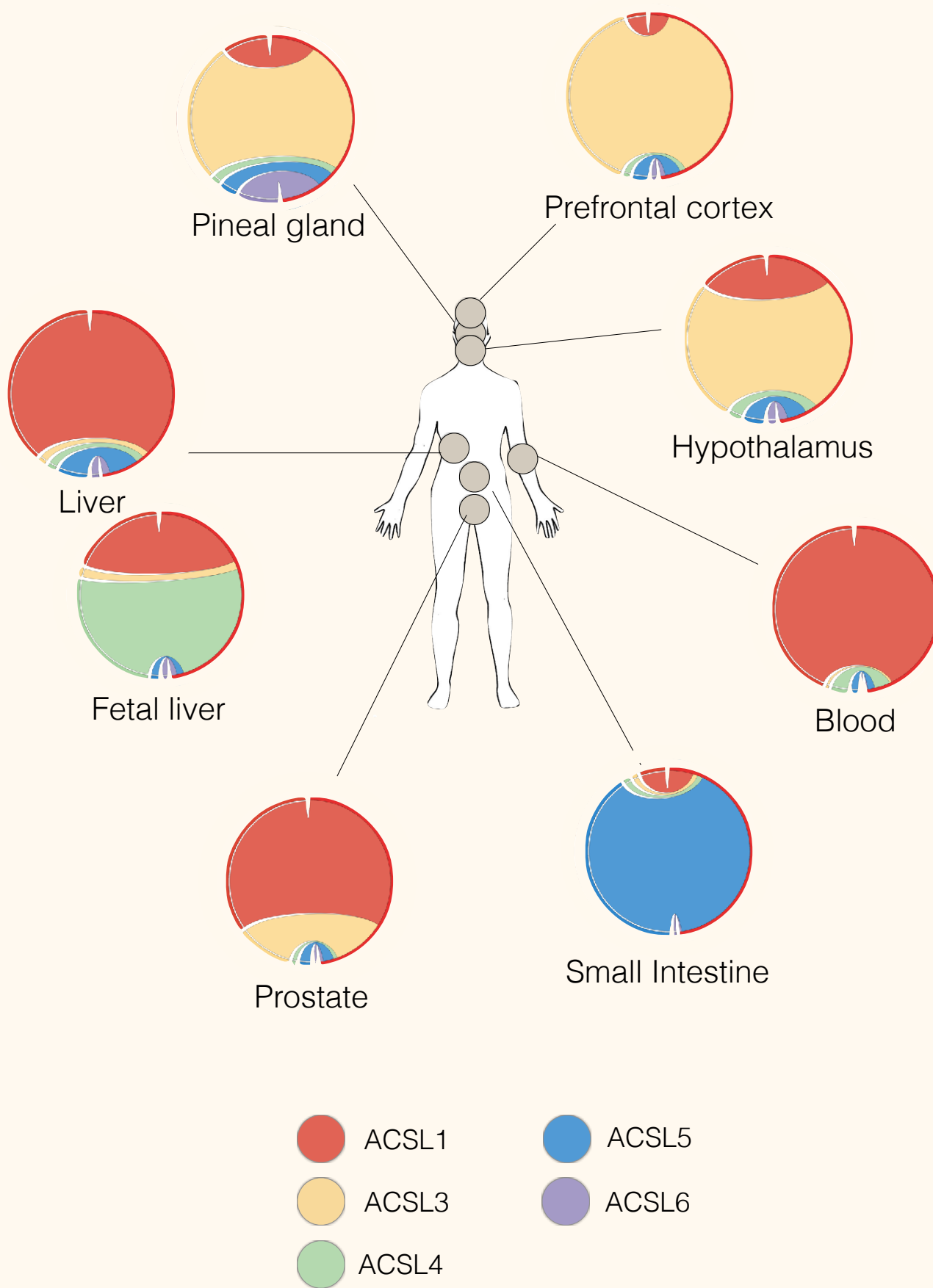

Supplementary Figure 1

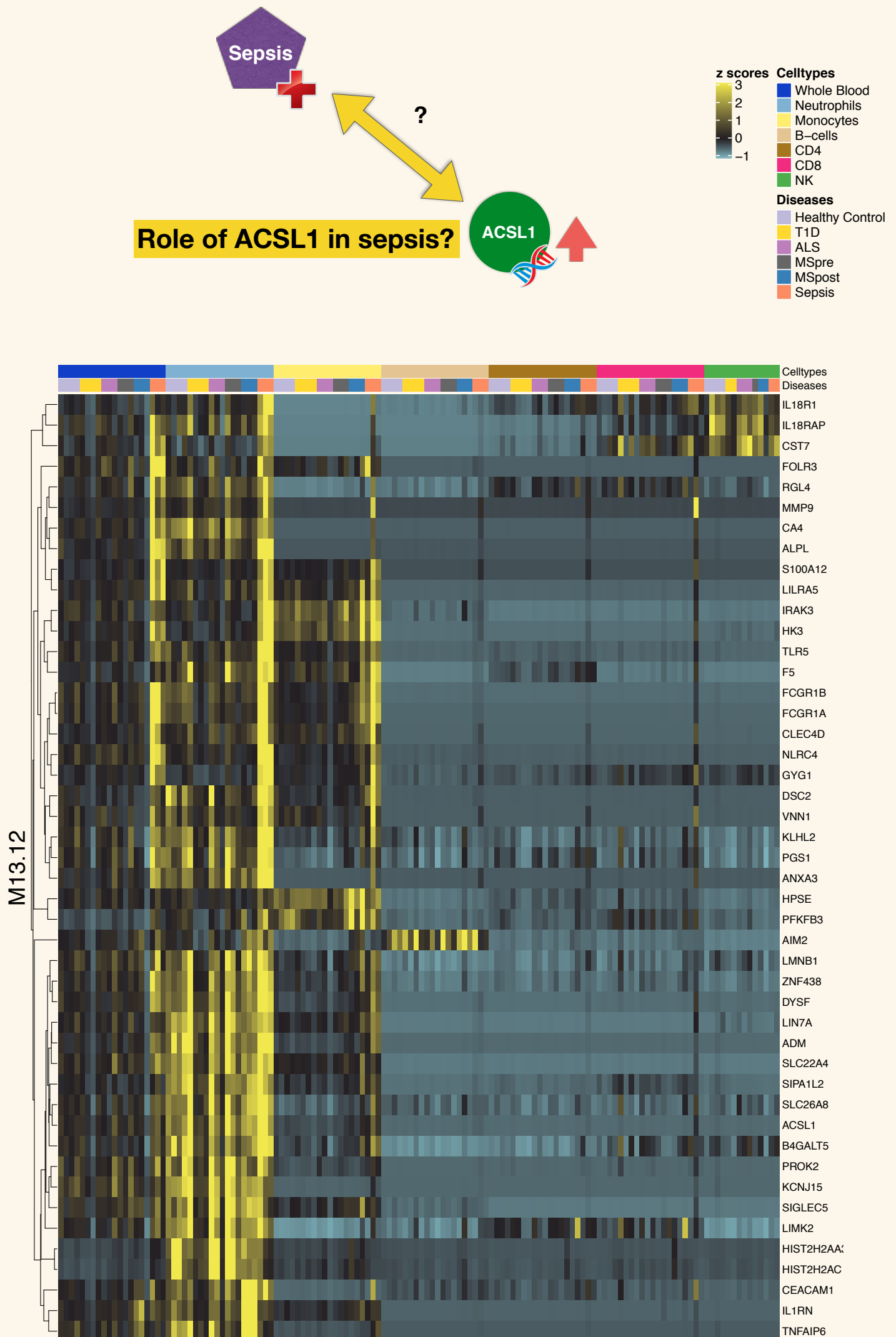

Supplementary Figure 2

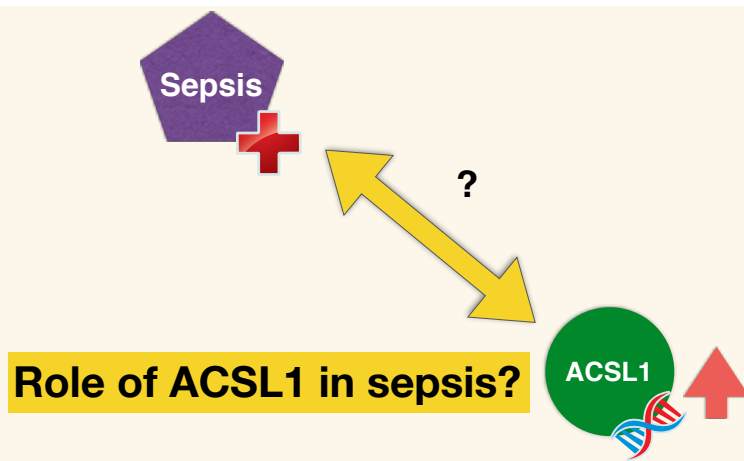

## Inflammasome

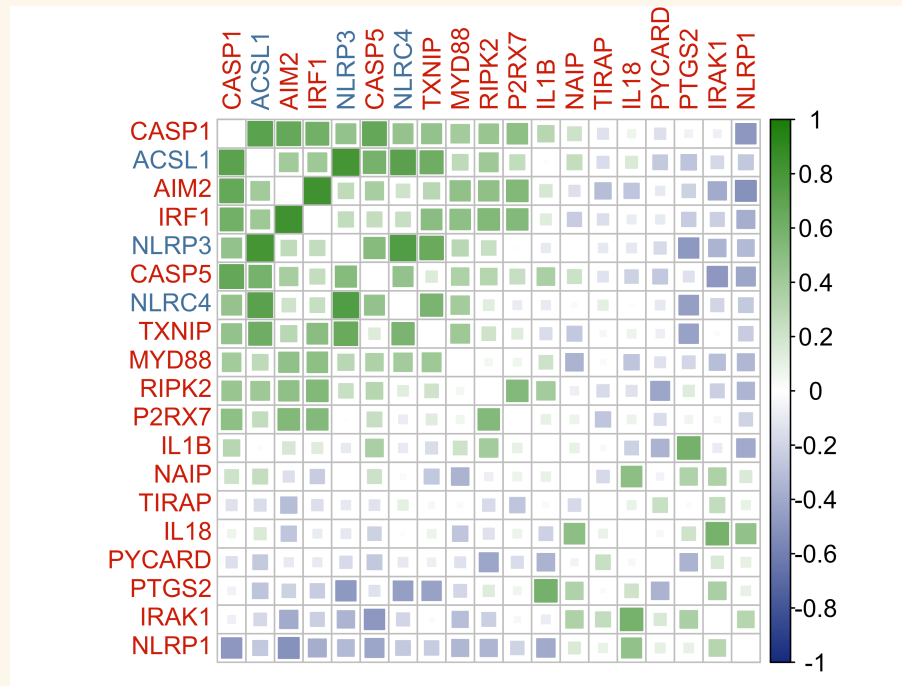

## Fatty acid metabolism

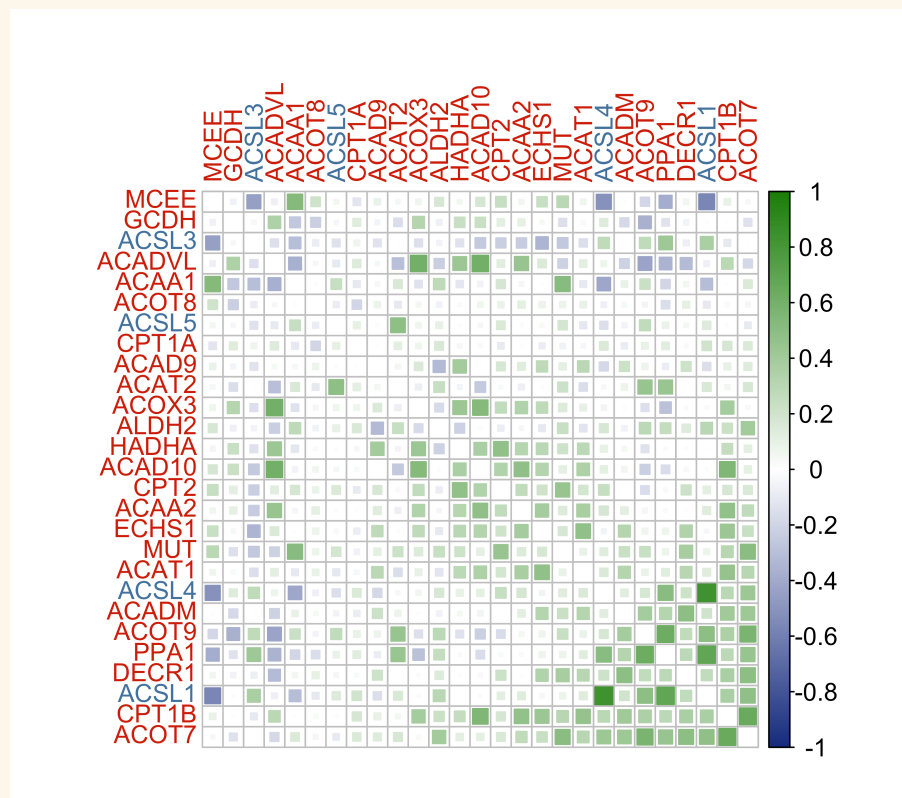

Supplement: Supplementary Figure 1 — Tissue restriction of ACSL family members. Relative abundance levels of the 5 members of the ACSL family across 8 different human tissues is shown is this figure. Expression data used to generate the plots were obtained from the BioGPS dataset (21). Each family member is represented by a different color: ACSL1 (red), ACSL2 (beige), ACSL4 (green), ACSL5 (blue), and ACSL6 (purple). Predominance of a given color in a circle denotes predominance of expression of the corresponding ACSL family member in the tissue in question. Cord plots were generated using the circlize R package (121). [file Presentation_1.pdf]
